# Molecular Mechanism of Arenavirus Assembly and Budding

**DOI:** 10.3390/v4102049

**Published:** 2012-10-10

**Authors:** Shuzo Urata, Jiro Yasuda

**Affiliations:** Department of Emerging Infectious Diseases, Institute of Tropical Medicine, Nagasaki University, 1-12-4 Sakamoto, Nagasaki 852-8523, Japan; Email: shuzourata@nagasaki-u.ac.jp

**Keywords:** arenavirus, assembly, budding

## Abstract

Arenaviruses have a bisegmented negative-strand RNA genome, which encodes four viral proteins: GP and NP by the S segment and L and Z by the L segment. These four viral proteins possess multiple functions in infection, replication and release of progeny viruses from infected cells. The small RING finger protein, Z protein is a matrix protein that plays a central role in viral assembly and budding. Although all arenaviruses encode Z protein, amino acid sequence alignment showed a huge variety among the species, especially at the C-terminus where the L-domain is located. Recent publications have demonstrated the interactions between viral protein and viral protein, and viral protein and host cellular protein, which facilitate transportation and assembly of viral components to sites of virus egress. This review presents a summary of current knowledge regarding arenavirus assembly and budding, in comparison with other enveloped viruses. We also refer to the restriction of arenavirus production by the antiviral cellular factor, Tetherin/BST-2.

## 1. Introduction

**Figure 1:**
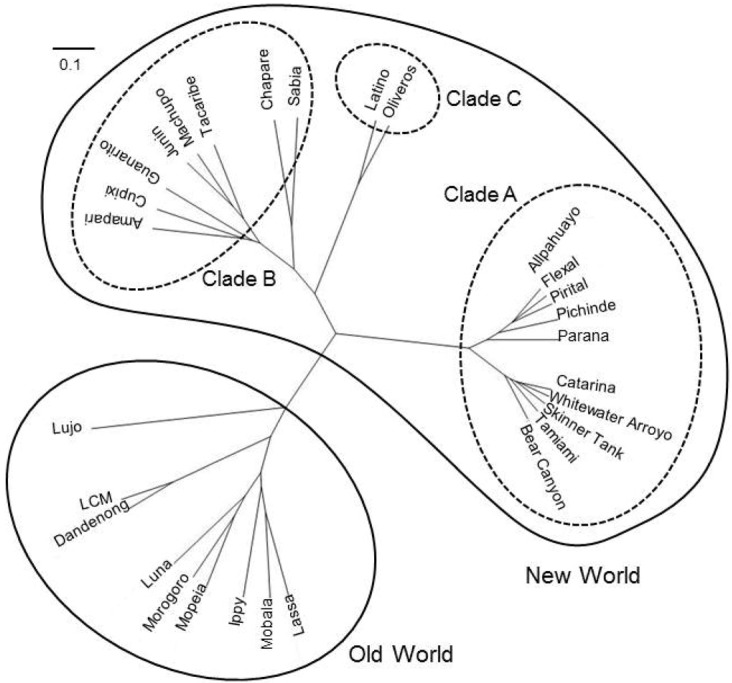
Phylogenetic tree of arenavirus Z protein. Amino acid sequences of Z protein were used for analysis. The phylogenetic tree was drawn using GENETYX [1]. The scale bar indicates substitutions per site. Accession numbers for reference sequences are: ABY20731 (Dandenong), YP_516232 (Ippy), NP_694871.1 (LASV, Josiah), ABC96003 (LCMV, Armstrong), YP_002929492 (Lujo virus), YP_516228 (Mobala virus), ABC71136 (Mopeia virus), YP_003090216 (Morogoro virus), YP_004933732 (Luna virus), YP_001649213 (Allpahuayo virus), AEQ59327 (Bear Canyon virus), AEQ59329 (Catarina virus), YP_001936023 (Flexal virus), YP_001936027 (Parana virus), YP_138535 (Pichinde virus), YP_025092 (Pirital virus), AEQ59336 (Skinner Tank virus), YP_001911119 (Whitewater arroyo virus), YP_001649217 (Amapari virus), YP_001816784 (Chapare virus), YP_001649219 (Cupixi virus), NP_899220 (Guanarito virus), NP_899216 (Junin virus, XJ-13), NP_899215 (Machupo virus), YP_089659 (Sabia virus), Q88470 (Tacaribe virus), YP_001911117 (Tamiami virus), YP_001936025 (Latino virus), YP_001649215 (Oliveros virus).

Arenaviruses are divided into two groups, Old World (OW) and New World (NW) arenaviruses, based on geographical, serological, and phylogenetic differences (Figure 1). 

Several arenaviruses cause hemorrhagic fever (HF) disease in humans. Lassa virus (LASV), Junin virus (JUNV), Machupo virus (MACV), Guanarito virus (GTOV), and Sabia virus (SABV) are the causative agents of Lassa fever (LF), Argentine HF, Bolivian HF, Venezuelan HF and Brazilian HF diseases, respectively [2]. In addition to these arenaviruses, Chapare virus and Lujo virus (LUJV) have also been reported to cause HF [3,4,5]. The prototypic arenavirus, Lymphocytic choriomeningitis virus (LCMV) has a world-wide distribution and is considered as a neglected human pathogen of clinical significance in congenital viral infections [6]. Moreover, LCMV infection of immunosuppressed individuals can result in severe disease and death [7,8]. These concerns are compounded by the lack of FDA licensed vaccines and limited existing therapeutic options. The live attenuated strain of JUNV, Candid #1, is the only arenavirus vaccine tested in humans, and has been licensed only in Argentina but is ineffective against LASV and LCMV. On the other hand, current arenavirus antiviral drug therapy is restricted to the use of the nucleoside analog ribavirin (Rib), which is only partially effective and is associated with significant side effects, including hemolytic anemia [9]. Therefore, there is now a pressing need for the development of effective anti-arenavirus therapeutic strategies. Several inhibitors of arenavirus entry have been reported [10,11,12,13,14,15,16,17]. The viral entry step is currently considered one of the most promising anti‑arenaviral targets. The success of Oseltamivir and Zanamivir, which are potent inhibitors of viral neuraminidase and prevent the release and spread of progeny virions, for the treatment of influenza clearly showed that the late steps of virus replication are also promising as antiviral targets. Recent studies have addressed the molecular mechanisms of virus budding. A number of studies yielded insight into the budding of enveloped viruses, including arenaviruses. However, this process seems to be more complicated than initially assumed. Despite of the complexity of the virus budding mechanism, there is evidence that targeting of viral budding and high-throughput screening (HTS) would be useful to identify specific anti-arenaviral drugs [18]. Utilization of arenaviral Z protein tagged with Gaussia luciferase (Gluc, 185 amino acid) at its C-terminus may be feasible for HTS [19]. This review presents a summary of current knowledge regarding arenavirus budding and the current model of arenavirus assembly and budding.

## 2. Molecular Biology of Arenaviruses

### 2.1. Arenavirus Genome Organization

Arenaviruses are enveloped viruses with a bisegmented negative-strand (NS) RNA genome and a life cycle restricted to the cell cytoplasm [2]. 

**Figure 2:**
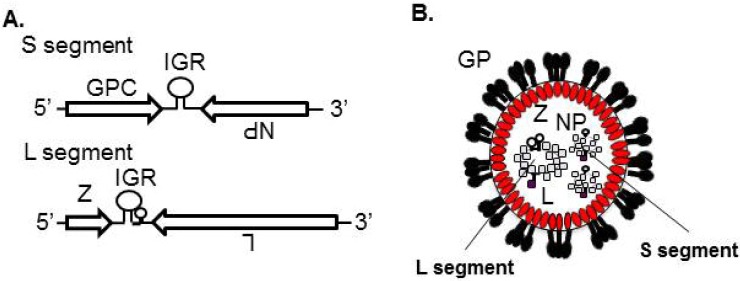
Genomic organization and virion structure of arenavirus. (**A**) Arenaviruses are enveloped viruses with a bisegmented NS RNA genome. Each genome segment uses an ambisense coding strategy to direct the synthesis of two viral polypeptides. The S segment encodes the NP and GPC. The L segment encodes the L and Z. (**B**) GP, which is posttranslationally processed by the cellular protease S1P/SKI-1, decorates the virion surface. Z lines the inside of the virion membrane. The viral genome encapsidated by NP and L is incorporated inside the virion. IGR: Intergenic region.

Each viral RNA (vRNA) segment, the small (S) segment or the large (L) segment, uses an ambisense coding strategy to direct the expression of two gene products in opposite orientation and separated by a non-coding intergenic region (IGR) (Figure 2A). The S segment (3.5 kb) encodes the viral nucleoprotein (NP) and the glycoprotein precursor (GPC), which is post-translationally processed to yield the peripheral virion attachment protein GP1 and the fusion-active transmembrane protein GP2. Trimers of GP1/GP2 (GP) form spikes that decorate the virus surface and mediate cell entry via receptor-mediated endocytosis (Figure 2B). The L segment (7.2 kb) encodes the L protein, an RNA‑dependent RNA polymerase, and the small zinc finger protein Z that is the viral matrix protein (Figure 2A,B).

### 2.2. Life Cycle of Arenavirus

**Figure 3:**
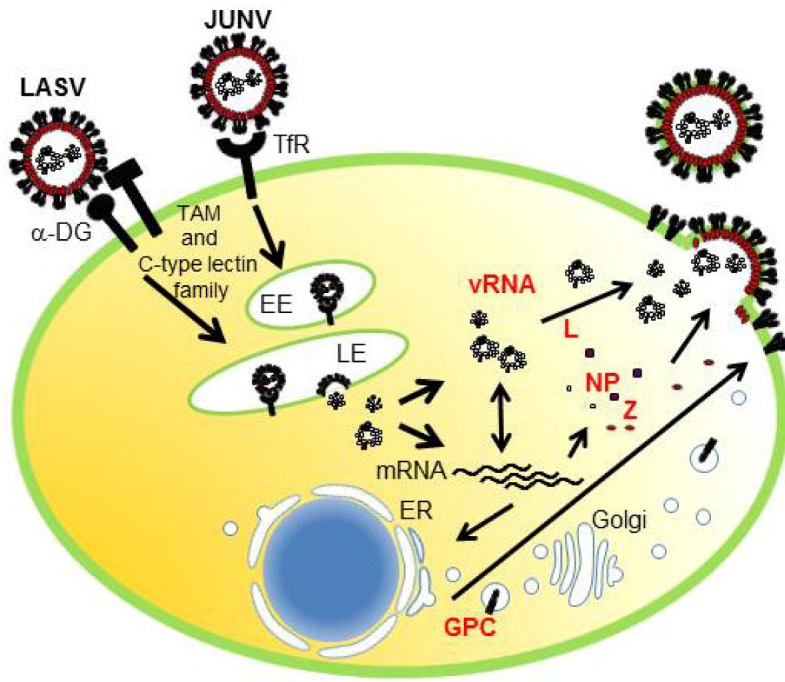
Life cycle of Arenavirus. LASV and JUNV use α-DG and TfR1, respectively, as the primary receptor. LASV also uses TAM family (Axl and Tyro3) and C-type lectin family (DC-SIGN and LSECtin) as receptors. JUNV enters the host cell by clathrin-mediated endocytosis, delivered to the Early endosome (EE) followed by the Late endosome (LE), while LASV enter cells by bypassing EE and reaches to the LE for the subsequent fusion process. Once vRNP is released into the cytoplasm, the viral genome is transcribed and replicated forvRNA and mRNA production. GPC is transported through the endoplasmic reticulum (ER) and Golgi compartments, where it is processed by S1P/SKI-1. Translated NP, L, and Z are transported to the platform for virus budding, gathered together with vRNP and cleaved GP, and then pinched off from the surface of infected cells.

Consistent with the broad host range and cell-type tropism, the highly conserved and widely expressed cell-surface protein α-dystroglycan (α-DG) has been identified as the main receptor for LCMV, LASV, and several other arenaviruses [20], whereas the human transferrin receptor 1 (TfR1) was identified as the primary receptor used by pathogenic clade B NW arenaviruses [21] (Figure 3). Recently, the TAM family (Axl and Tyro3) and C-type lectin family (DC-SIGN and LSECtin) were also reported to function as receptors for LASV [22] (Figure 3). Following receptor binding, the virions are internalized through the clathrin-independent or clathrin-dependent endocytotic pathway for OW or NW arenaviruses, respectively [23,24]. Interestingly, entry of OW LCMV and LASV into the cell is independent of caveolin, dynamin, actin, and the small GTPases, Rab5 and Rab7, but dependent on cholesterol [23,24]. Following release of the viral ribonucleoprotein (vRNP) into the cytoplasm of infected cells, the vRNP-associated viral RNA polymerase initiates viral RNA replication and gene transcription. Release of infectious progeny virions from the infected cell involves association of the vRNP core and surface GP complex along with viral matrix protein Z (Figure 3).

## 3. Role and Function of Arenavirus Z Protein

### 3.1. Structure and Function of Z Protein

**Figure 4:**
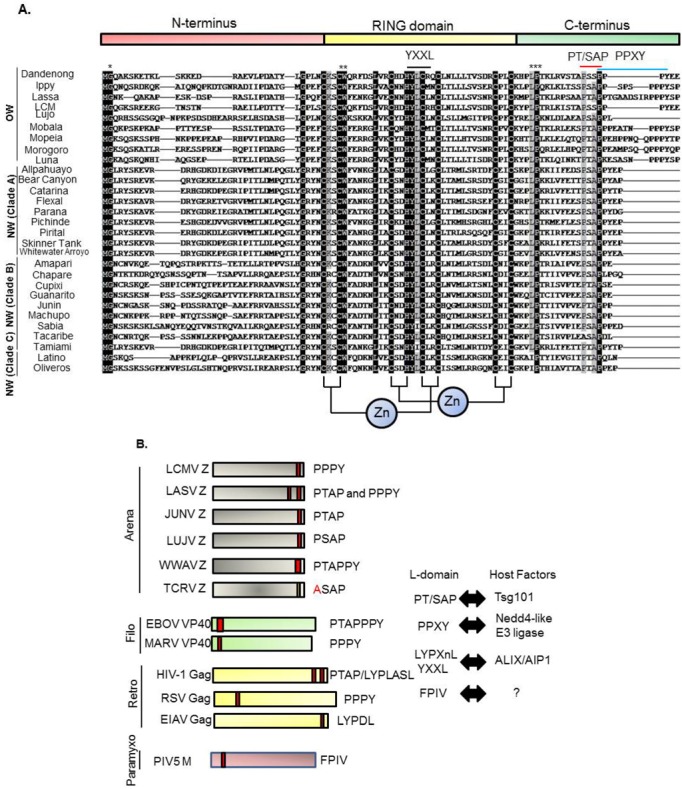
Alignment of amino acid sequences of arenavirus Z proteins. (**A**) The Z proteins are arranged by clade using ClustalW. N-terminus, central RING domain, C-terminus as well as L-domains are indicated above. Arenavirus clades are indicated on left. Conserved zinc motifs are indicated below the alignment. * shows conserved myristoylation site glycine 2 (G2), ** tryptophan (W), *** proline (P). Black box indicates 100% match and gray box indicates 90% match of amino acid among the Z proteins. (**B**) L-domains within matrix proteins of enveloped viruses. L-domains and their interacting host factors are indicated. Accession numbers for reference sequences are described in the legend of Figure 1.

NP and L have been shown as the minimal components for efficient vRNA synthesis using mini‑genome (MG)-based assays [25]. Z has been shown to inhibit both viral RNA replication and transcription using arenaviral MG systems [26,27,28,29,30,31]. The function of Z in the replication and transcription were described in recent reviews in detail [18,32]. In brief, the Really Interesting New Gene (RING) domain of Z was strictly required, but not sufficient, for this inhibitory effect [26] (Figure 4A). The RING domain of Z mediates an interaction with some cellular proteins, including the promyelocytic leukemia protein PML and the translation initiation factor eIF4E. The interaction between LASV Z and PML inhibited the function of eIF4E by decreasing the binding affinity of eIF4E to the m(7)G cap ligand [33]. However, the biological roles of these interactions on arenavirus replication are still largely unknown. It might be worth to note that overexpression of PML had little effect on LCMV replication [34]. Recently, viral RNA synthesis and gene expression of MACV was reconstituted *in vitro*. The study using this system showed that the binding of Z to L impaired the catalytic activity of L [35]. In addition to the inhibitory role of Z in transcription and gene expression, NW arenavirus Z have been shown to bind to RIG-I and inhibit IFN-β activation [36]. 

NMR structure of the LASV Z RING domain showed that Z interacts directly with eIF4E [33]. It has been shown that type I IFN production by plasmacytoid dendritic cell (pDC) plays an important role on innate immune responses and interferon regulatory factor 7 (IRF7) regulates this IFN production. Since the translation of IRF7 in pDC is influenced by the eIF4E-BP/eIF4E ratio, it is of interest to examine the effect of Z on the IFN production in pDC [37]. 

### 3.2. Comparison of Z Protein Sequences among Arenavirus Family

As described above, Z protein is a viral matrix protein possessing bona fide budding activity. The Z protein consists of three domains, *i.e.*, the N-terminal domain, central RING domain, and the C‑terminal domain (Figure 4A). The function of the N-terminus has not been well characterized. In all the arenavirus Z proteins, glycine at position 2 (G2) is myristoylated and is important for attaching to the plasma membrane (PM) (Figure 4A). The role of Z protein myristoylation is also described in section 5 (“Roles of phosphatidylinositol (PI) at the PM, myristoylation of viral matrix protein, and PI3K on viral budding”). The central region of Z protein contains the RING domain, which is well‑known to function in E3 ligase as well as protein-protein interactions [38,39]. As described above, the best characterized function of the RING domain in Z is the regulation of viral gene expression [26,27,35]. 

It is not known whether this RING domain contributes to Z-mediated budding by itself. It has been shown that the YLCL motif in the Mopeia (MOPV) Z RING domain recognizes ALIX/AIP1 and recruits NP [40,41]. The best characterized domain in Z is the C-terminus where the L-domain is located (Figure 4A,B). The L-domain is a short amino acid motif, which plays an essential role in virus budding, and three motif sequences, PT/SAP, PPXY, and YPXnL (or YXXL), have been characterized [42]. In addition, other sequences including FPIV were also reported to act as an L-domain [43]. Although the cellular host factors that interact with many of these L-domains have been identified [42,44,45], the cellular partner of the FPIV sequence has not been found (Figure 4B). 

Three L-domain motifs, PT/SAP, PPXY, and YPXnL (or YXXL), interact with the cellular proteins, Tsg101, Nedd4-like E3 ligase, and ALIX/AIP1, respectively [42]. Tsg101 and ALIX/AIP1 are components of the Endosomal Sorting Complex Required for Transportation (ESCRT) complex. The ESCRT machinery is involved in protein sorting, cytokinesis, and autophagy, and all of which are key mechanisms for cellular homeostasis [46]. We describe the interactions between L-domains and cellular factors in detail in section 6 (“Involvement of ESCRT machinery in the budding of enveloped viruses”). 

As shown in Figure 4A,B, the Z proteins of OW arenaviruses possess PT/SAP (or similar to PT/SAP) and PPPY, but LUJV possesses only the PTAP motif. Some NW arenaviruses also possess only the PT/SAP motif. PICV and WWAV possess overlapping PT/SAPPY, very similar to that of Ebola virus (EBOV) VP40 [47]. Tacaribe virus (TCRV) Z does not possess a canonical L-domain, but rather possesses an L-domain-like motif. We and others previously showed that the TCRV Z ASAP motif (similar to the PT/SAP motif) do not contribute to Z-mediated budding [48,49]. It will be important to assess the different budding modes among arenaviruses and their functional mechanisms.

### 3.3. Z as a Viral Matrix Protein

Viral matrix proteins of most RNA viruses, including retroviruses, possess a bona fide budding activity. That is, sole expression of viral matrix protein can produce virus-like particles (VLPs). This makes it possible to examine the molecular mechanism of virus budding, especially for BSL-4 agents, such as LASV. In this regard, HIV-1 Gag is the best characterized matrix protein. Initially, the p6 domain of HIV-1 Gag was reported to play a critical role in HIV-1 budding [50,51,52]. Other retrovirus Gag proteins have also been characterized in detail, including equine infectious anemia virus (EIAV) [53], Moloney murine leukemia virus (Mo-MLV) [54], Mason-Pfizer monkey virus (M-PMV) [55], Rous sarcoma virus (RSV) [56,57], prototypic foamy virus (PFV) [58], and human T-cell leukemia virus type 1 (HTLV-1) [59]. Vesicular stomatitis virus (VSV) and Rabies virus (RV) M and EBOV and Marburg virus (MARV) VP40 also function as viral matrix proteins [47,60,61,62,63,64,65,66,67,68,69]. The M proteins of paramyxoviruses, which includes measles virus [70,71], Newcastle disease virus (NDV) [72], SV5 [43], human parainfluenza virus (hPIV) [73], Sendai virus [74], and Nipha virus [75,76,77], have also been shown to have budding activity [78,79] (Figure 4B). In the case of arenaviruses, we and others have shown that LASV and LCMV Z are viral matrix proteins [80,81,82,83]. Z proteins of JUNV, MACV, TCRV, and MOPV have also been reported to be viral matrix proteins [40,48,49,84,85]. All Z proteins of these viruses possess G2, central RING domain, and L-domain at the C-terminus [18] (Figure 4A). 

### 3.4. Domain Requirement for Arenavirus Z-Mediated Assembly and Budding

Although Z plays a central role in arenavirus assembly and budding, little is known about the domain(s) required for this process. The best studied domain is the L-domain, which is described above. In addition, G2 of Z has been shown to be myristoylated similar to HIV-1 Gag, which facilitates its membrane anchorage (Figure 4A). Several studies have indicated the importance of phosphatidylinositol for the association of HIV-1 Gag with PM [86,87]. It is not yet known whether the same mechanism is crucial for arenavirus budding. Capul *et al.* demonstrated that the highly conserved amino acid residues among the arenavirus Z proteins are important for incorporation of vRNP into the virion, but not budding itself [88]. 

It was shown that myristoylation of TCRV Z at G2 is important for self-assembly of Z proteins [89]. This suggests that self-assembly of Z occurs once Z interacts with the PM through its myristoylation. In addition, R37, N39, W44, L50, and Y57 (Zinc domain I) in TCRV Z were shown to be critical for the interaction with viral L protein [90]. TCRV Z also interacts with NP, and this role in viral budding is described in section 4.1. (“Transport of vRNA and NP to the budding site”) [49,89]. LCMV and LASV Z interact with the C-terminus of NP, which does not overlap with the self-assembly domain and the functional domain as an interferon (IFN) antagonist [91,92]. These results indicated that this Z‑NP interaction recruits vRNP complex and induces uptake into infectious progeny virions. Interestingly, it was also shown that there is some specificity of NP-Z interaction between LASV and LCMV [91]. Briefly, LCMV NP interacts with both LASV and LCMV Z, but LASV NP interacts only with LASV Z. A previous study showed that JUNV Z L79, which is conserved among almost all arenaviruses, is important to recruit TCRV NP into Z-mediated VLP and its interaction facilitates GP incorporation into VLP [85] (Figure 4A). 

### 3.5. Role of the L-Domain in Virus Replication and Pathogenesis

Previously, it was reported that the requirement of L-domain of HIV-1 Gag for virus propagation was cell type dependent [93]. Targeting of HIV-1 Gag to PM or Multi-vesicular body (MVB) and the L-domain requirement for virus budding seem to be cell type dependent [93,94,95,96]. In the case of RV, the PPXY motif is critical for its replication and pathogenesis in a mouse model [97]. In the case of VSV, which belongs to the *Rhabdoviridae* along with RV, virus budding in BHK-21 cells is dependent on the PPPY motif, but not PSAP, within M protein [98]. The PPPY motif in VSV M was also shown to be important for VSV replication on HEK293T cells [99]. These studies also implied that Vps4A/B requirements may be different among cell types for VSV replication. In the case of EBOV, the L-domain is not essential for replication and propagation in cell culture, although the L-domain mutant virus showed a 2–3 log reduced in viral titer compared to wild-type (WT) virus in Vero cells [100]. It has not been determined whether the role of L‑doomain on arenavirus replication and budding are cell type dependent. However, the data from other enveloped viruses containing multiple L-domain motifs suggest that the requirement for the L‑domain located in the C-terminus of Z for efficient replication and budding may depends on cell type.

## 4. Intracellular Transport of Other Viral Components

### 4.1. Transport of vRNA and NP to the Budding Site

All the virion components must be concentrated at the site of budding, but the mechanism underlying this process is largely unknown for arenaviruses. The presence of IGR in vRNA, which is a hairpin structure aligned between two viral coding genes in both L and S segments, is one of the features of arenavirus [2] (Figure 2A). IGR plays a critical role in LCMV genome incorporation [101]. Previous studies showed that Z interacts with NP, L, and GP [40,49,85,89,90,91,102]. Through the interaction between Z and NP/L, vRNP may be recruited into the virion (Figure 8).

The contribution of NP to LCMV and LASV Z-mediated budding has not been known, although LCMV and LASV NP interact with LCMV and LASV Z, respectively. On the other hand, some arenaviruses’ NP were reported to contribute to the assembly and budding processes. One example is MOPV NP. ALIX/AIP1, one of the ESCRT components, can interact with both MOPV NP and Z, and therefore bridges the interaction between NP and Z [41] (Figure 4A). This interaction facilitates MOPV Z-mediated budding. Another example is TCRV NP. TCRV Z-mediated budding is enhanced by the co-expression of TCRV NP, which was not observed with JUNV Z and NP [49]. These observations suggest that arenavirus Z protein may utilize a different mechanism to facilitate budding. 

Although we previously showed that Tsg101 is important for MARV VP40-induced VLP budding and the PPPY motif located in VP40 is crucial for Tsg101-mediated budding and incorporation into VP40-induced VLP [66], it is unlikely that MARV VP40-Tsg101 interaction is direct [103] (Figure 4B). MARV NP was reported to interact with Tsg101, and facilitate VP40-induced VLP production [104]. Based on these data, it is possible that viral proteins can recruit ESCRT components with several different mechanisms and facilitate budding.

### 4.2. Transport of GP to the Plasma Membrane

**Figure 5:**
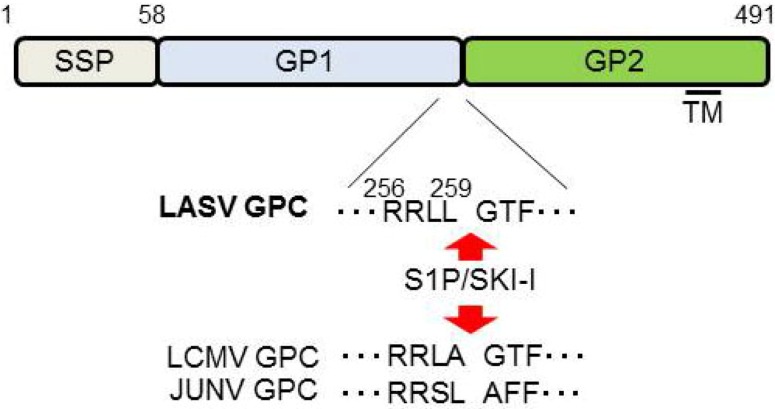
Schematic representation of LASV GPC. Cleavage sites for S1P/SKI-1 are indicated by the red arrows. The sequences of LCMV and JUNV cleavage sites are also indicated. SSP: stable signal peptide; TM: Transmembrane domain.

GP is a viral surface glycoprotein that is responsible for binding to the cellular receptor and for the subsequent fusion event (Figure 3). LASV, LCMV [20], as well as clade C of NW arenaviruses [105] use α-DG as the primary receptor, whereas clade B of NW arenaviruses utilize TfR1 [21,106] (Figure 3). Therefore, the incorporation of GP into the virion is a critical process to produce infectious progeny virions. GPC is cleaved by a cellular site 1 protease (S1P/SKI-1) at either ER or Golgi [107,108,109,110], to form GP trimer complex [108,111] (Figure 5). Interestingly, this GP cleavage by S1P/SKI-1 is necessary to incorporate GP into virions, but not transportation to the cell membrane [23]. Recently, it was shown that the S1P/SKI-1 processing is different between GPC and cellular substrates, such as SREBP2 and ATF6, based on the dependency on S1P/SKI-1 autoprocessing [110]. This S1P/SKI-1 cleavage is also important for GP oligomerization [112]. Thus GPC cleavage by S1P/SKI-1 is an attractive target for arenavirus therapy [113,114,115,116,117]. Indeed, we and others have reported that the small chemical compound PF-429242, which targets S1P/SKI-1, can be a leading compound to combat arenavirus [115,118]. In addition, stable signal peptide (SSP) located at the N-terminus of GPC was shown to play a critical role in GPC trafficking of LCMV and JUNV [119,120] (Figure 5), as well as its own expression, processing, cis-acting, and fusion activity [121,122]. SSP was also showed to interact with myristoylated Z [102]. The N-glycosylation of GP is also important for its expression, cleavage, and fusion activity, and these influence virion infectivity [123,124]. 

## 5. Roles of Phosphatidylinositol (PI) at the PM, Myristoylation of Viral Matrix Protein, and PI3K on Viral Budding

PIs are lipids distributed in the membrane that are highly regulated by both phosphatases and kinases. Phosphatidylinositol (4, 5) (PI (4, 5) P2) is the most abundant, and is specifically localized on the cytoplasmic leaflet of the PM, and catalyzed by class I PI3Ks to generate PI (3, 4, 5) P3. PI (4, 5) P2 was shown to act as a platform for HIV-1 budding [87,125,126]. The monomeric HIV-1 Gag was proposed to interact with PM through PI (4, 5) P2, thus triggering a conformational change in Gag, followed by myristoylation of G2 [127,128]. This HIV-1 Gag myristoylation has been characterized in detail [129]. It has also been shown that HIV-1 Gag is myristoylated by N-myristoyltransferase (NMT)-1 and -2 [130]. In addition, pH [131] and calmodulin (CaM) binding to the Gag MA domain [132,133] are also important for Gag myristoylation. Furthermore, RNA has been proposed to be required for this Gag-PM binding [134]. Once Gag is myristoylated at PM, Gag-Gag multimerization occurs, and increases the strength of PM-Gag interaction [135,136]. Similarly, oligomerization of TCRV Z is also dependent on its myristoylation [90]. As arenavirus Z is also myristoylated [48,137,138], it is likely that PI acts as a platform for Z-PM interaction. Recently, we demonstrated that Phosphatidylinositol 3-kinase (PI3K) is involved in arenavirus budding [118]. In this study, we used the pan class I PI3K inhibitor, LY294002, as well as dual PI3K/mTOR inhibitor, BEZ-235, which is currently in clinical trial as an anti-solid tumor drug [139]. Based on our results, mTOR seems not to be involved in this activity. Which class of PI3K contributes and the underlying mechanism remain to be elucidated. Nonetheless, we showed that targeting of PI3K can be a novel approach to combat arenavirus infections. 

## 6. Involvement of ESCRT Machinery in the Budding of Enveloped Viruses

**Figure 6:**
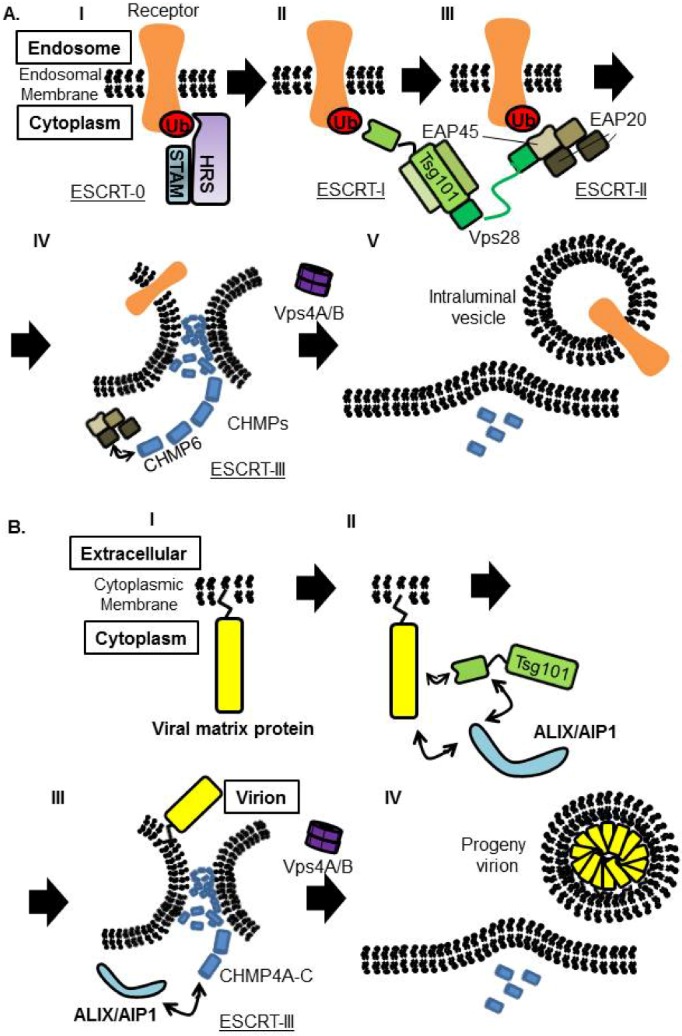
Proposed model of receptor sorting and virus budding. (**A**) Model of receptor sorting. Ubiquitinated receptor is recognized by ESCRT-0, followed by Tsg101, one of the ESCRT-I components. ESCRT-II is proposed to connect ESCRT-I and ESCRT-III. ESCRT‑III and the AAA ATPase Vps4A/B trigger the membrane scission to form intraluminal vesicle inside the endosome. (**B**) Viral matrix protein attached to PM and is recognized by Tsg101. Tsg101 binds to ALIX/AIP1 directly, and ALIX/AIP1 also binds to CHMP4, members of the ESCRT-III components to induce vesicle formation.It is not fully understand how ESCRT-I and ESCRT-III is connected in the virus budding procedure. ESCRT-III complex is disassembled by Vps4A/B, for recycling and initiation of new rounds of sorting or budding.

The ESCRT pathway exists in all eukaryotes and consists of six heterooligomeric complexes (ESCRT-0, ESCRT-I, ESCRT-II, ESCRT-III, ALIX/AIP1, and VPS4A/B). These complexes are recruited sequentially to membranes and function in protein sorting, membrane remodeling, membrane fission, *etc.* [46,140]. The ubiquitinated endosomal cargo is first recognized and bound by HRS in ESCRT-0 complex at the membrane, resulting concentration of ubiquitinated cargo on the endosomal membranes (Figure 6AI). In the next step, the cargo is bound by ESCRT-I, through Ubiquitin E2 Variant (UEV) domain of Tsg101, and ESCRT-II is recruited to the membrane. These ESCRT-I and ‑II complexes together induce bud formation (Figure 6AII,III). Finally, ESCRT-III complex mediates membrane scission from the cytosolic side of the bud. ESCRT-III is disassembled and recycled by the VPS4A/B/LIP5 complex (Figure 6AIV,V). 

Since the discovery of the interaction between Tsg101 and HIV-1 Gag, and that this interaction facilitates HIV-1 budding, many of the enveloped viruses have been shown to utilize the ESCRT pathway to bud from the cell. The PT/SAP motif within viral matrix protein recognizes the UEV domain of Tsg101 and ride on the ESCRT pathway. The importance of Tsg101 in viral budding has been reported for a variety of viruses [42,44,141]. Interestingly, VSV M possess PT/SAP motif, but neither Tsg101 nor Vps4 are required for replication [142]. 

ESCRT-II, which contains two copies of EAP20 and a single copy each of EAP30 and EAP45, interacts with both ESCRT-I and -III (Figure 6AIII). However, it has been shown that depletion of EAP45 by RNAi did not affect either HIV-1 budding or cytokinesis [143]. This result indicated that ESCRT-II may have a specific function only in MVB vesicle formation (Figure 6).

Although Tsg101 is the most important factor to initiate budding when viral matrix protein possesses the PT/SAP motif, interaction with ALIX/AIP1, which also bridges Tsg101 and CHMP4, can overcome Tsg101 deficiency [144,145] (Figure 6B). EIAV Gag does not possess the PT/SAP motif, but the YPDL motif in EIAV Gag binds directly to the V domain of ALIX/AIP1 and facilitates its budding [144] (Figure 4B and Figure 6B). It was also reported that ALIX/AIP1 played an important role in bridging MOPV Z and NP [40,41]. We showed previously that ALIX/AIP1 does not contribute to LASV Z-mediated budding based on analysis using siRNA specific for ALIX/AIP1 [82], but we did not examine whether ALIX/AIP1 contributes to other processes.

ESCRT-III is comprised on charged MVB proteins (CHMPs) (Figure 6). In humans, twelve proteins were reported to compose the ESCRT-III complex. These ESCRT-III proteins play a critical role in membrane fission by assembling into helical filaments within the membrane neck structure. Recently, it was shown that utilization of these CHMPs is regulated in a sophisticated manner between HIV-1 budding and cytokinesis, as only some are required for HIV-1 budding, but most play a significant role in cytokinesis [146]. 

Vps4 is an AAA-ATPase, and involved in disassembly and recycling of ESCRT-III complex. Vps4 is compromised by Vps4A and Vps4B, and binds to LIP5, which serves as an activator of Vps4 assembly and ATPase activities (Figure 6).

HCET-type Nedd4-like E3 ligases are recognized to bind to the PPXY motif of viral matrix protein through its WW domain (Figure 4B). The WW domain consists of 38–40 amino acids containing two widely spaced tryptophans (W) and binds to a variety of proteins containing the PPXY motif. Although E3 ligase does not belong to ESCRT components, many studies have demonstrated the importance of E3 ligase in viral budding. Nonetheless, the precise roles of these E3 ligases in the viral budding process are still unknown. It is obvious that there is some specificity of this WW domain to bind and regulate PPXY motif-mediated budding, as the specific WW domain acts in a dominant negative manner for the budding. We showed previously that the Nedd4.1 WW domain does not inhibit LASV Z-mediated budding [82], but this WW domain inhibited EBOV and MARV VP40‑mediated budding [147,148]. Gag proteins of many retroviruses, including HTLV-1, RSV, MoMLV, and M‑PMV, have been shown to interact with specific E3 ligases and be regulated [149,150,151,152,153,154,155]. Noteworthy, overexpression of Nedd4.2 (or Nedd4L) enhanced its budding, although HIV-1 Gag does not possess canonical PPXY motif [156,157], suggesting an important role of E3 ligase on HIV-1 replication. 

LASV Z possesses the PPPY motif which is critical for virus budding [81]. However, the E3 ligase which specifically regulates the LASV budding has not been identified. The Arrestin-related trafficking (ART) proteins have been reported to bridge ESCRT machinery and E3 ligases. Arenaviral Z proteins, which possess PPXY motif such as LASV, might be connected to the ESCRT machinery via ART proteins, together with the PTAP motif-Tsg101 interaction [45,158].

## 7. Regulation of Viral Assembly and Budding by IFN

### 7.1. Regulation of Virus Budding by Interferon-Stimulated Genes (ISGs)

IFN is a well-characterized host immune molecule that triggers expression of hundreds of so-called interferon stimulated genes (ISGs). In general, many of the viruses encode specific proteins that antagonize innate immune responses [159,160]. The tripartite motif containing 5α (TRIM5α) has been shown to be induced by IFN-α, and is well known as a HIV-1 entry host restriction factor [161,162,163]. In addition, TRIM5α has been shown to regulate HIV-1 budding [164,165]. Recent reports have suggested that not all of the ISGs show anti-viral activity, but rather exhibit specificity among viruses [166,167]. A wide scale experiment was performed to determine the antiviral activities of ISGs against hepatitis C virus, yellow fever virus, West Nile virus, chikungunya virus, Venezuelan equine encephalitis virus, and HIV-1. The authors screened more than 380 ISGs independently to determine their antiviral activities against these selected viruses, and demonstrated the diversity of these functions [166]. A total of 288 ISGs were then tested against VSV and murine gammmaherpes virus 68 (MHV-68) [167]. It is not clear whether these ISGs contribute to inhibition of arenvirus assembly, budding, and release. Therefore further analysis is required.

### 7.2. The Function of Ubiquitin or Ubiquitin-Like Proteins (UBL) on Arenavirus Budding

Ubiquitin is a molecule of 76 amino acids with multiple functions. Ubiquitin binds to the substrate, normally at lysine (K), and the substrates are either mono- or poly-ubiquitinated. Monoubiquitination is involved in intracellular trafficking of the substrates. On the other hand, poly-ubiquitination leads to substrate degradation via the ubiquitin-proteasome pathway to maintain cell homeostasis. Although ubiquitin is not an IFN-inducible protein, one of the ubiquitin-like proteins (UBL), ISG15 has been shown to regulate viral assembly and budding. Previously, it was reported that HIV-1 Gag and EBOV VP40 are tagged with ISG15, and regulates the budding process [168,169]. In addition, ISG15 was reported to be tagged to ESCRT III components, CHMP2A, CHMP4B, CHMP5, and CHMP6 [170,171,172]. Interaction of Tsg101 with ubiquitinated HIV-1 Gag was reported to be 10-times stronger than that with non-ubiquitinated Gag [173]. Therefore, ubiquitin and ISG15 have been suggested to be important for viral budding, but the precise role of these in arenavirus budding is unclear [174]. Some studies utilized proteasome inhibitors to assess the importance of ubiquitin, but this treatment seems to affect multiple cellular processes, not only viral protein ubiquitination and viral budding [174]. 

### 7.3. Inhibition of Virus Release by the Interferon-Induced Cellular Protein, Tetherin

As described above, some interferon-induced proteins clearly show antiviral activity specifically at the late stage of virus replication, which included ISG15 and TRIM5α [164,165,168,169]. Recently, one of these ISGs, named Tetherin (also known as BST-2, CD317, or HM1.24), has been reported as an inhibitory cellular factor against HIV-1 [175,176]. We and other groups reported that Tetherin shows antiviral activity against other retroviruses, filoviruses, and arenaviruses [177,178]. Tetherin is constitutively expressed in terminally differentiated B cells, bone marrow stromal cells, and pDC, and is also broadly induced by treatment with Type I and Type II IFNs in various cell types [176]. Therefore, Tetherin is thought to be involved in antiviral host defense as one of the innate immunity mechanisms. In addition, Tetherin expression has been shown to be increased in multiple myeloma [179], endometrial cancer [180], primary lung cancer [181], and glioma cells [182]. Therefore, several groups have attempted immunotherapy using anti-Tetherin antibodies, which mediate antibody-dependent cellular cytotoxicity [181,183].

**Figure 7:**
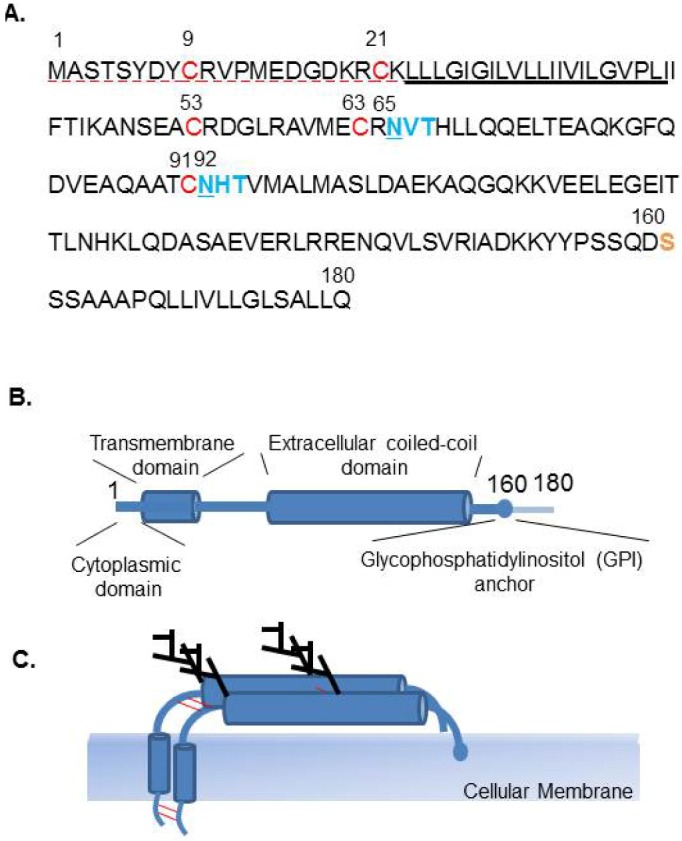
Characteristics of human Tetherin. (**A**) Amino acid sequence of human Tetherin (accession number NP_004326). Dashed red underline (1-22) indicates the cytoplasmic domain (CD), underline indicates the transmembrane domain (TM). Red font (C9, C21, C53, C63, and C91) indicates amino acids that may be responsible for disulfide bond formation and dimerization. Blue font indicates potential N-linked glycosylation motif (N‑X-S/T) and site (underlined). Serine (S) at position 160 is indicated as the site of cleavage prior to the C-terminal addition of a GPI anchor in yellow font. (**B**) Schematic representation of the domain structure of Tetherin, a type II transmembrane protein. (**C**) Tetherin forms a homodimer, through a C-C disulfide bond. Two sites of N‑glycosilation are also indicated.

Tetherin is a type II transmembrane protein, consisting of four domains, *i.e.*, an N-terminal cytoplasmic domain (CD), a single transmembrane domain (TM), an extracellular domain, and a putative C-terminal glycosyl phosphatidylinositol (GPI) anchor (Figure 7A–C), which is present on the cell surface and in perinuclear compartments. Tetherin is anchored in the cell membrane at both ends, and combined high-resolution crystallography and small-angle X-ray scattering-based modeling indicated that the Tetherin ectodomain forms a parallel coiled-coil homodimer [184,185,186,187,188]. Therefore, it has been suggested that Tetherin inhibits progeny virus release by directly tethering virions to cells, briefly by anchoring one end of the molecule on the cell membrane and the other end on the viral envelope. Progeny virions released from cells could also be directly tethered to each other by Tetherin. Electron microscopy was performed to confirm this model, and based on the distances between tethered virions and cells, the membrane-spanning model was proposed as the model of action of Tetherin [176,189,190]. Interestingly, an artificial Tetherin-like protein, composed by domain replacement of TfR at the N-terminus, dystrophia myotonica protein kinase (DMPK) at the ectodomain, and urokinase plasminogen activator receptor (uPAR) at the C-terminus was also shown to have antiviral activity similar to that of Tetherin, suggesting that this typical conformation or configuration acts to tether virion progeny at the cell surface [189]. 

Some viruses encode IFN antagonists to avoid or inhibit the innate immune response, such as arenavirus NP, EBOV VP35, and influenza virus NS1 [159,191,192]. In general, viral antagonists inhibit the innate immune signaling cascades. In addition to these IFN antagonists, some viruses encode specific viral proteins that antagonize the antiviral activity of Tetherin [178,193]. HIV-1 Vpu is the best characterized Tetherin antagonist. HIV-2 Env, SIV Env, SIV Nef, KSHV K5, and EBOV and MARV GP have also been reported as Tetherin antagonists, but their mechanisms of action seem to differ among these viral proteins [178,193,194,195]. 

Initially, we reported that Tetherin inhibits LASV Z-mediated VLP release, and this restriction was not overcome by LASV GP, but was overcome by HIV-1 Vpu expression [177]. In addition, we showed that the glycosylation of Tetherin at 65 and 92 asparagine (N) is dispensable for its activity [177]. Moreover, we showed that Tetherin dimerization through its C-C interaction had little effect on its inhibitory activity against Lassa VLP release, but was not essential for its antiviral activity [196]. It is likely that tethering of virions by Tetherin dimers is stronger than that by Tetherin monomers because of the stronger association with the membrane. Therefore, Tetherin appears to inhibit release of a wide variety of enveloped viruses from host cells by a similar mechanism, as Tetherin shows antiviral activities against many retroviruses, filoviruses, arenaviruses, and KSHV. However, there is controversy regarding the importance of glycosylation and dimerization of Tetherin for its antiviral activity.

Recent studies using mouse models have assessed the importance of Tetherin with regard to its antiviral activity against Mo-MLV [197], mouse mammary tumor virus (MMTV) [198], VSV, and influenza B virus [199]. In case of Mo-MLV and MMTV, knock out (KO) or knock down (KD) of Tetherin exhibited higher titer of these viruses *in vivo*, suggesting Tetherin exhibited antiviral activity against these viruses. In addition, HIV-1 Vpu, which antagonizes Tetherin, has been shown to contribute to the efficient spread of HIV-1 using the humanized mouse model transplanted human hematopoietic stem cells [200]. On the other hand, KO of Tetherin showed lower titer of VSV and influenza virus B [199]. These results indicated that the antiviral effect of Tetherin *in vivo* is complicated and showed different phenotype depending on the virus species. 

In the case of arenaviruses, one study demonstrated that the infectious LASV was inhibited by human Tetherin [84]. In addition, this report indicated that LASV does not possess any Tetherin antagonist [84]. It is not yet clear whether Tetherin has any effect on arenavirus propagation and pathogenesis *in vivo*. 

## 8. Arenavirus Assembly and Budding Model

**Figure 8:**
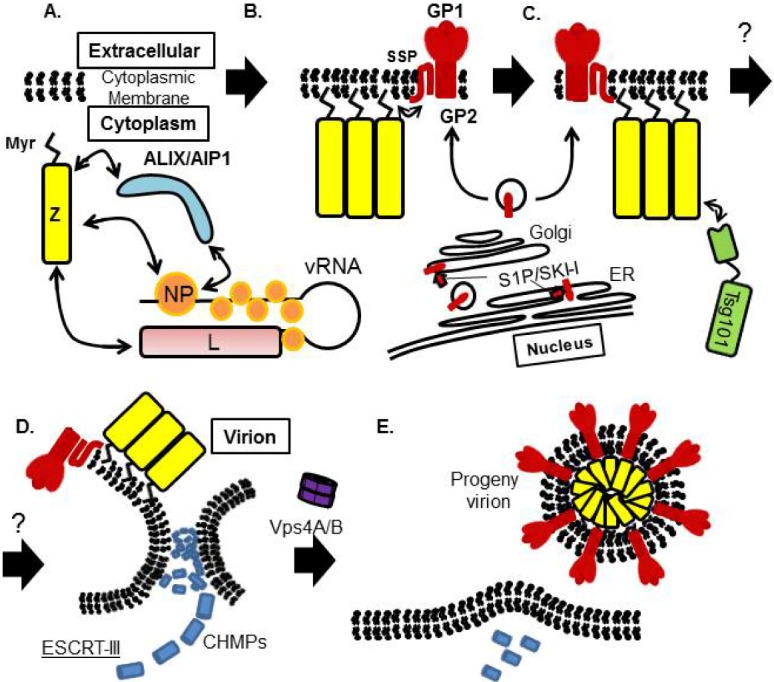
Proposed model for arenavirus budding. (A) Z interacts with NP and L, which encapsidates vRNA to compose vRNP complex. MOPV Z interacts with NP through ALIX/AIP1. (**B**) Once Z is myristoylated, Z interacts with PM, and formed multimerization. Myristoylated Z was shown to interact with SSP of GP, which was cleaved by S1P/SKI-1 and transported to PM. (**C**) Z interacts with Tsg101 to initiate budding processes. (**D**) Vesicle is formed and Z is layered as an inner leaflet and cleaved GP is incorporated, decorate outside the virion. (**E**) Membrane is scissor by the function of ESCRT-III and Vps4 complex, and the progeny virion is pinched off from the infected cell.

Taking the model for regulation of arenavirus RNA synthesis by Z together with the results of NMR analysis, it is proposed that at the early stage of arenavirus replication, when Z is present at low concentration, Z permits ongoing RNA synthesis. Once Z reaches a high concentration, the binding of Z to RNP results in inhibition of viral transcription and replication. Transportation of Z to the cellular membrane (endosomal membrane or PM) is not known well. Studies from TCRV suggested that Z-Z assembly occurs after attaching to the PM. Therefore, Z might interact with NP and L, which encapsidate vRNA to compose vRNP complex, before interaction with PM (Figure 8A). Upon transportation of Z to the site of budding together with vRNP, Z interacts with PM through its myristoylation and may shift its conformation allowed the multimerization (Figure 8B) and the binding to Tsg101 (Figure 8C). Myristoylation of Z also allows the interaction with SSP of GP (Figure 8B). When Z is forming the membrane curvature orchestrating with ESCRT components, GP that has already been cleaved by S1P/SKI-1 at either ER or Golgi can be incorporated into the virion (Figure 8B–D). It is not fully understood how Tsg101 connects to ESCRT-III in the virus budding procedure. Finally, the progeny virus is pinched off from the cells upon ESCRT-III and Vps4 activation (Figure 8E).

## 9. Concluding Remarks

This review described current knowledge regarding arenavirus assembly and budding. Although many groups have been worked in this field, many questions remain unanswered, including the role of E3 ligase in the budding process, identification of ISGs that contribute to (or inhibit) assembly and budding, as well as the precise molecular mechanisms by which these proteins facilitate virus budding. 

Novel arenaviruses have been identified on average every three years. It is easy to imagine that a new highly pathogenic arenavirus may emerge, similar to Lujo virus [4]. Although individual arenaviruses use different strategies for assembly and budding, they can be classified into a few groups based on alignment of the Z C-terminus. Therefore, investigation of the molecular mechanism underlying this assembly and budding processes, and understanding of the common and unique mechanisms are necessary to develop and identify new antiviral therapies to combat pathogenic arenaviruses. 
